# Flame retardant exposure assessment: findings from a behavioral intervention study

**DOI:** 10.1038/s41370-018-0049-6

**Published:** 2018-06-28

**Authors:** Elizabeth A. Gibson, Heather M. Stapleton, Lehyla Calero, Darrell Holmes, Kimberly Burke, Rodney Martinez, Boris Cortes, Amy Nematollahi, David Evans, Julie B. Herbstman

**Affiliations:** 1 Department of Environmental Health Sciences, Mailman School of Public Health, Columbia University, New York, NY 10032 USA; 2 Nicholas School of the Environment, Duke University, Durham, NC USA; 3 Department of Pediatrics, College of Physicians & Surgeons, Columbia University, New York, NY 10032 USA

**Keywords:** Flame retardants, intervention, polybrominated diphenyl ethers, organophosphate flame retardants

## Abstract

**Background:**

Polybrominated diphenyl ethers (PBDEs) have been largely replaced by organophosphate flame retardants (OPFRs) and alternative brominated flame retardants (Alt-BFRs) to meet flammability requirements. Humans are ubiquitously exposed to some variety of flame retardants through contact with consumer products directly or through household dust.

**Objectives:**

To evaluate the effectiveness of house cleaning and hand washing practices to reduce exposure to flame retardants, we measured concentrations in dermal hand wipes and urinary metabolites before and after assignment to two consecutive interventions.

**Methods:**

We selected 32 mother and child dyads from an existing cohort. This analysis focuses on mothers. Participants provided baseline measurements (urine, hand wipes, and questionnaires) and were then assigned for 1 week to either a house cleaning (including instruction on proper technique and cleaning supplies) or hand washing (including instruction on proper technique and soaps) intervention arm. For the second week, participants were assigned to the second intervention in addition to their initial assignment, thus all subjects both washed their hands and cleaned according to the intervention guidelines during week 2. We collected measurements at the end of weeks 1 and 2.

**Results:**

We found reductions in urinary analytes after week 1 of house cleaning (BCIPHIPP and ip-DPHP), week 1 of hand washing (BCIPP, BCIPHIPP, and tbutyl-DPHP), and week 2 of combined interventions (BCIPHIPP and tbutyl-DPHP), compare to baseline. We found no significant decline in hand wipes in the entire sample but did find reductions after week 1 of house cleaning (BDE 209), week 1 of hand washing (TCEP), and week 2 of combined interventions (TDCIPP and BDE 209) in women with exposure above the median at baseline (verified through simulations).

**Conclusions:**

Exposure to individual flame retardants was reduced by about half, in some cases, by 1 week of increased hand washing, house cleaning to reduce dust, or combined activities.

## Introduction

Anthropogenic flame retardant chemicals are included in a myriad of consumer products, ranging from polyurethane foam in furniture to electronics, to meet the flammability requirements at the state and federal levels in the US. [1, 2]. Many of these additive flame retardants are not chemically bound to consumer products, and thus have a tendency to migrate into the external environment [3]. Polybrominated diphenyl ethers (PBDEs) were originally the most highly used chemicals for reducing flammability in furniture because of their low cost, efficiency, and availability [4, 5]. Growing concerns over the health impacts of exposure (i.e., neurobehavioral effects) [6–11] led to the voluntary phase-out of industrial production and application of penta- and octa-BDE mixtures by 2005. Phase-out of deca-BDE was meant to be completed by 2013, with the largest producers and importers of deca-BDE in the US committing to end its production, importation, and sale for all uses, but its status is still uncertain [12–14].

To replace these PBDE mixtures [4, 15, 16], manufacturers have introduced organophosphate flame retardants (OPFRs) and alternative brominated flame retardants (Alt-BFRs) which have increased in use since 2005. OPFRs (also referred to as PFRs (phosphorous flame retardants) and OPEs (organophosphate ethers)), including triphenyl phosphate (TPHP), tris(1,3-dichloroisopropyl) phosphate (TDCIPP, also called Tris), tris-(2-chloroethyl) phosphate (TCEP), and tris(1-chloro-2-propyl) phosphate (TCIPP), have become pervasive in the environment and in humans [17–22]. OPFRs are used not only as flame retardants, but also in other applications, such as plastics [23]. Two Alt-BFRs, 2-ethylhexyl-tetrabromobenzoate (TBB) and bis(2-ethylhexyl) tetrabromophthalate (TBPH), components of a commercial mixture known as Firemaster550 (FM 550) [4], which also contains OPFRs, have been repeatedly detected in household dust [15, 24] and at varying levels in human urine (from 27% detection [25] to 77% detection [26]). Dust is believed to be the primary pathway of exposure. Since flame retardants are not chemically bound to consumer products, they leach into the external environment and are inadvertently ingested through dust exposure, primarily via hand-to-mouth activity [27]. OPFRs are rapidly metabolized, with half-lives of several hours in animal models, compared to half-lives of PBDEs between 1.8 and 6.5 years in humans [3, 20, 28–32]. However, due to continuous exposure to both OPFRs and Alt-BFRs through household products, exposure measures likely approximate a constant body burden, with intra-class correlations (ICCs) of 0.50 (DPHP) and 0.72 (BDCIPP) previously reported [26, 33].

OPFRs are structurally similar to neurotoxic organophosphate pesticides and have demonstrated neurotoxicity in laboratory models, raising concerns about exposure and toxicity to humans [34–37]. In human epidemiologic studies, OPFR exposure has been associated with disruptions of the endocrine system [17, 38], decreased fertility [39], and thyroid function [40]. More research is needed to understand the toxicokinetics and potential effects of Alt-BFRs [41].

The US Environmental Protection Agency (EPA), in assessing the risks of flame retardant exposure, provides recommendations for exposure mitigation to parents of young children [42]. This advice to minimize exposure to flame retardants is based on previous research implicating dust as the principle exposure pathway of PBDEs [27]. The EPA suggests practical steps, including hand washing, especially before eating, and house cleaning, specifically dusting with a moist cloth, wet mopping, and vacuuming, to reduce exposure to flame retardants [42].

In the present study, we assessed changes in urinary flame retardant metabolite levels and in dermal concentrations measured by hand wipes before, during, and after a population-based behavioral intervention study based on EPA recommendations designed to mitigate exposure to flame retardants through household dust.

## Materials and methods

### Participants

We selected 32 mother and child dyads from the previously established Sibling-Hermanos Cohort, which began in 2008, consisting of Dominican and African–American mothers and children from Northern Manhattan and the South Bronx. Briefly, these women were enrolled in the Columbia Center for Children’s Environmental Health Mothers and Newborns birth cohort between 1998 and 2006 [43], and when subsequently pregnant with a singleton, they were invited to enroll an additional child in the Sibling-Hermanos cohort, which was followed prospectively [24]. Among participants with children between the ages of 3–6 years between December 2015 and May 2016, we invited women and children from the Sibling-Hermanos Cohort to participate in our intervention study, described in detail below.

The current analysis of this intervention is restricted to mothers because they provided more complete samples and data than children. In an ancillary report, we describe the relationship between flame retardants measured in paired samples from mothers and children (in preparation).

### Intervention

**Fig. 1:**
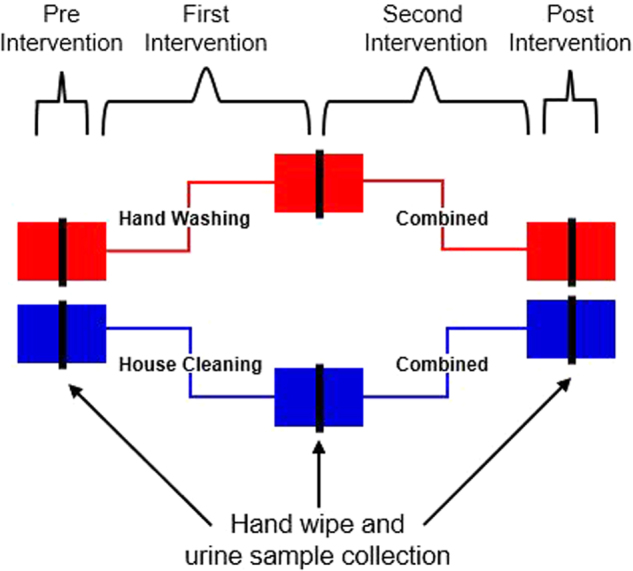
Intervention design

Interventions were based on EPA recommendations for flame retardant exposure mitigation [42]. All participants provided urine and hand wipe samples and completed a detailed questionnaire at baseline. Participants were randomly assigned to one of two study arms, conditional on race/ethnicity, to guarantee equal distributions of African–American and Dominican women in each arm. This ensured that race/ethnicity, which has been found to be associated with flame retardant exposure [44], was not associated with intervention arm. Those in the cleaning arm were told to clean their home with an emphasis on removing dust during week 1, given instructions on proper cleaning techniques, and incentivized with flame retardant-free cleaning products, mops and buckets, microfiber dust cloths, and handheld vacuums without a HEPA filter. They were asked to use the vacuum as much as they liked, with the suggestion that they open windows while vacuuming to reduce exposure to recirculating dust. Those in the hand washing arm were told to wash their hands in a specific manner during week 1, given instructions on proper washing techniques and necessary length of time, and incentivized with flame retardant-free soaps. They were asked to wash their hands more often, particularly before they ate. Directions and reinforcement materials are included in the Supplemental materials. All participants provided urine and hand wipe samples and completed a follow-up questionnaire after week 1 (Fig. 1).

During week 2, all participants were assigned to both study arms. Those who cleaned during week 1 were additionally instructed to increase hand washing during week 2. Those who washed their hands during week 1 were additionally instructed to increase house cleaning during week 2. All participants provided urine and hand wipe samples and completed a follow-up questionnaire after week 2 (Fig. 1). With this crossover design, all participants had equivalent assignments at baseline (pre-intervention) and after week 2 (combined intervention), but assignment at week 1 varied with equal groups of 16 families assigned to each arm.

### Sample collection

All adults gave informed consent for themselves and their children before sample collection. At each visit (baseline, after week 1, after week 2), we administered a short questionnaire to the mothers which included information about hours typically spent in the home and typical hand washing and house cleaning behaviors. At each visit, we collected a spot urine sample and a hand wipe sample from the mother and her child. As previously described [45], we wiped the entire palm and back surface of both hands from the base of the fingernails to the wrist with a 3×3 pre-cleaned cotton pad saturated with 3 mL of isopropyl alcohol. We wrapped the collected hand wipe in an aluminum foil packet, inserted it into a glass vial, and covered the glass vial in bubble wrap, which was subsequently stored in a cooler for transport to our laboratory where samples were stored at −20 °C. We collected field blanks (whose purpose was to assess the potential for field contamination) at 10% of randomly selected households by saturating a pre-cleaned wipe with isopropyl alcohol and placing it directly into an aluminum foil packet.

### Laboratory analysis

Hand wipe and urine samples were extracted and analyzed using methods published previously for each matrix for PBDEs, OPFRs, and Alt-BFRs in hand wipes and for OPFRs in urine [20, 22, 25, 26]. Entire hand wipe samples were spiked individually with F-BDE-69, 13C-BDE-209, 13C-TBB, and 13C-TPHP as internal standards (positive controls) and extracted three times by sonication with 1:1 hexane/acetone (v/v). The combined extracts were concentrated (roughly 45 mL) to 1 mL using a nitrogen evaporation system and transferred to an autosampler vial for gas chromatography/mass spectrometry analysis. F-BDE-69, 13C-BDE-209, 13C-TBB, and 13C-TPHP recoveries averaged 94.39%, 55.36%, 84.66%, and 71.19%, respectively.

We used a digital hand-held refractometer (Atago) to measure specific gravity for each urine sample. Using methods described previously, 5.0 mL of urine was spiked individually with d-BDCIPP, d-DPHP, and d-TDCIPP as internal standards and combined with a sodium acetate buffer and an enzyme solution, then incubated overnight at 37 °C. The flame retardant metabolites were extracted via mixed-mode anion-exchange solid-phase extraction and measured using atmospheric pressure chemical ionization liquid chromatography–tandem mass spectrometry (Agilent Technologies, Model 6410) [22]. d-BDCIPP, d-DPHP, and d-TDCIPP recoveries averaged 107%, 69.4%, and 69.1%, respectively. The method detection limits (MDLs) were calculated using three times the standard deviation of the blanks normalized to the volume of urine extracted. Six urinary metabolites of OPFR flame retardants were measured: bis(1,3-dichloro-2-propyl) phosphate (BDCIPP), bis(1-chloro-2-propyl) phosphate (BCIPP), bis(1-chloro-2-isopropyl) 1-hydroxy-2-propyl phosphate (BCIPHIPP), diphenyl phosphate (DPHP), two alkylated DPHPs (ip-DPHP and tbutyl-DPHP) (Table 1). MDLs ranged from 0.08 ng/mL (ip-DPHP and tbutyl-DPHP) to 0.64 ng/mL (BCIPHIPP) for urinary metabolites. All hand wipes and urine samples were analyzed at Nicholas School of the Environment, Duke University.

**Table 1:** Relationships between measured analytes on hand wipes and metabolites in urine

Parent compound on hand wipe	Urinary metabolite
TDCIPP	BDCIPP
TPHP	DPHP
TCIPP	BCIPP
	BCIPHIPP
TCEP	Not measured
Not measured	ip-DPHP
Not measured	tbutyl-DPHP
PBDEs	Not measured
Alt-BFRs	Not measured

### Data analysis

Urinary concentrations are reported as analyte mass per volume (nanograms per milliliter (ng/mL)), normalized by specific gravity to account for urinary dilution [46]. Hand wipe concentrations are reported as total analyte mass per hand wipe (nanograms (ng) from both hands). We examined the concentration and distribution of flame retardant analytes in each sample. Measurements below the MDL were assigned the sample-specific MDL/√2 [47]. All statistical analyses were repeated separately for urine and hand wipe measurements.

We measured 29 analytes in hand wipes, including 23 PBDEs, two Alt-BFRs, and four OPFRs, and six OPFR metabolites in urine. Analyses were conducted for analytes with detection frequency >50%. We summed five representative PBDEs (BDE 47, BDE 99, BDE 100, BDE 153, and BDE 154) and both measured Alt-BFRs (TBB and TBPH) to create two composite scores, ΣBDE and ΣAlt-BFR, for concentrations in hand wipes. This analysis includes ΣBDE, BDE 209, ΣAlt-BFR, TCEP, TCIPP, TDCIPP, and TPHP in hand wipes and all urinary metabolites of OPFRs (relationships between parent compounds and metabolites are detailed in Table 1).

Flame retardant concentrations were not normally distributed; as they approximated a log-normal distribution, we log-transformed all concentrations in regression models and used non-parametric tests for correlation and differences between time points (sensitivity analysis). We conducted bivariate analyses using linear regression with log-transformed flame retardant concentrations as the outcomes to determine if baseline exposure concentrations differed by demographic variables or by baseline cleaning practices. Because this study included seven analytes in hand wipes and six metabolites in urine, consistency in direction and magnitude were assessed across flame retardants within each sampling matrix to identify predictors of flame retardant exposure. Variables found to be associated with baseline concentrations of flame retardants and also associated with intervention arm despite random assignment (e.g., hours spent at home) were included as covariates in statistical models.

We used mixed-effects models for repeated measures (three urine samples and three hand wipes within each individual), with participant included as a multilevel random effect to evaluate the changes in concentrations over time. Intervention was categorized into four groups—baseline, week 1/cleaning, week 1/hand washing, and week 2/combined—and this variable was included as a fixed effect to assess the changes from baseline to the end of week 1 for each intervention arm and from baseline to the end of week 2 for the combined study sample. We adjusted all hand wipe models for time since last hand wash and hours spent at home and all urine models for hours spent at home (during the previous week).

We conducted sensitivity analyses on these results using the Wilcoxon signed-rank test on matched pairs to assess the differences in concentrations between baseline and week 1 within study arms and between baseline and week 2.

After stratifying the mixed-effects models by participant’s exposure at baseline (categorized as either high or low based on the median concentration), we repeated the analysis for all flame retardants. Because of concerns over regression to the mean when focusing on participants with initially high exposure, we simulated a null association across the course of the study (i.e., no change from baseline) within those with high baseline exposure levels. To do this, we sampled from the baseline distribution of those 16 individuals above the median exposure level (individuals classified as high varied across flame retardants), with replacement, three times to create three time points within individuals to simulate a model of null association. We repeated this sampling and modeling process 1000 times. We then took the beta coefficients from the bootstrapped mixed-effects models (with components identical to the original models) to create a distribution of coefficients representative of random chance. We finally compared our observed coefficient from the mixed-effects models to the betas generated from the random distribution. We repeated these steps for the 16 individuals below median exposure at baseline. In the instances where our observed coefficient was greater than two standard deviations from the mean of the randomly generated distribution of beta coefficients, we affirmed that our finding could not be explained as a byproduct of regression.

We evaluated the influence of individual participants on exposure. To evaluate the effects over the course of the study, we estimated the ICC within individuals (the variance attributable to the random effect of each participant divided by the total variance).

We also compared the measurements between flame retardant levels in hand wipes and urine. We calculated Spearman correlation coefficients to examine the associations between urinary metabolites and their parent compounds in hand wipes, averaging baseline and week 1 samples to evaluate the correlation during week 1’s intervention, and averaging week 1 and week 2 samples to evaluate the correlation during week 2’s shared intervention, and creating overall composites of urine samples and of hand wipes (the average of three time points, each) to evaluate correlation across the length of the study.

We performed statistical analyses in SAS statistical software (version 9.4; SAS Institute Inc., Cary, NC) and in R (version 3.3.3; R Development Core Team 2017); statistical tests were conducted at the 0.05 significance level.

## Results

Thirty-two mothers provided baseline urine and hand wipe samples. Of these, two mothers failed to provide urine samples and one failed to provide a hand wipe sample after week 2. The mean age of mothers in the study was 32.6 years. The intervention arms differed marginally on average hours at home per day (*p* = 0.05). Characteristics of study participants are provided in Table 2.

**Table 2:** Characteristics of study participants at baseline

Variable		Intervention group		
	Overall^a^	Hand washing^a^	House cleaning^a^	*p* ^b^
	*n*=32	*n*=16	*n*=16	
Race/ethnicity				0.72
African American	12 (37.50)	5 (31.25)	7 (43.75)	
Dominican American	20 (62.50)	11 (68.75)	9 (56.25)	
Maternal age (years)		32.72 (3.76)	32.42 (4.59)	0.84
Avg times hands washed/day				0.53
1–2	2 (6.25)	0 (0.00)	2 (12.50)	
3–5	11 (34.38)	5 (31.25)	6 (37.50)	
6–8	8 (25.00)	4 (25.00)	4 (25.00)	
9+	11 (34.38)	7 (43.75)	4 (25.00)	
Avg hours/day spent in home				0.053
1–2	2 (6.25)	0 (0.00)	2 (12.50)	
3–4	10 (31.25)	3 (18.75)	7 (43.75)	
5–6	7 (21.88)	3 (18.75)	4 (25.00)	
7+	13 (40.63)	10 (62.50)	3 (18.75)	
Maternal education				0.64
Some high school	12 (37.50)	7 (43.75)	5 (31.25)	
High school degree or equivalent	15 (46.88)	8 (50.00)	7 (43.75)	
Some college	3 (9.38)	1 (6.25)	2 (12.50)	
College degree	2 (6.25)	0 (0.00)	2 (12.50)	

^a^Values are mean (standard deviation) or number (%)

^b^*p*-Values are from *t* test or Fisher’s exact test for differences between intervention arms

DPHP, BDCIPP, and ip-DPHP were detected in 100% of maternal urine samples at baseline (Table 3). BCIPHIPP and tbutyl-DPHP were both detected in 96.9% of urine samples. BCIPP was detected in 87.5% of maternal samples. We found detectable levels of all analytes in 100% of hand wipes. Geometric means (with standard deviations) in maternal urine samples at baseline ranged from 0.22 (1.94) ng/mL for tbutyl-DPHP to 6.92 (2.16) ng/mL for ip-DPHP. Geometric means for analytes in hand wipes at baseline ranged from 11.75 (2.50) ng for BDE-209 to 167.82 (3.82) ng for TPHP (Table 3).

**Table 3:** Pre-intervention concentrations of parent compounds in hand wipes and urinary metabolites

		Mothers (*n* = 32)							
						Percentiles			
	MDL^a^	G Mean	G Std dev	# <MDL	% <MDL	25th	50th	75th	Max
*Hand wipes*									
TCEP (ng)	2.70	29.17	2.14	–	–	15.65	30.2	57.93	111.84
TCIPP (ng)	19.80	224.83	2.63	–	–	103.06	218.91	466.77	2969.79
TDCIPP (ng)	3.90	133.75	2.63	–	–	76.41	127.34	244.37	1557.57
TPHP (ng)	1.10	167.82	3.82	–	–	76.71	131.42	252.59	4965.36
ΣAlt-BFRs (ng)	0.17	47.52	2.82	–	–	21.675	33.735	96.31	440.33
ΣBDEs (ng)	0.14	38.74	2.47	–	–	20.465	33.42	60.985	330.33
BDE 209 (ng)	0.12	11.75	2.50	–	–	6.345	11.21	21.915	73.56
*Urine samples*									
BCIPP (ng/mL)	0.15	0.82	3.31	4	12.5	0.36	0.76	1.93	11.57
DPHP (ng/mL)	0.45	3.34	2.48	–	–	1.90	2.76	4.45	39.75
BDCIPP (ng/mL)	0.19	1.06	2.41	–	–	0.65	1.11	2.04	7.77
BCIPHIPP (ng/mL)	0.64	1.27	2.22	1	3.1	0.75	1.35	2.18	6.89
ip-DPHP (ng/mL)	0.08	6.92	2.16	–	–	4.72	7.77	10.72	33.05
tbutyl-DPHP (ng/mL)	0.08	0.22	1.94	1	3.1	0.15	0.22	0.33	1.24

^a^The reported MDL for composite measures (TBB + TBPH and ΣBDEs) is the lowest MDL of the sum

### Differences in exposure at baseline

Demographic variables (race/ethnicity—Dominican or African American, maternal education, and age), cleaning practices (frequency of cleaning, type of cleaning, window position (opened/closed) during cleaning, and frequency of hand washing), and lifestyle factors (number of stuffed pieces of furniture at home, nail biting, diet, and hours spent at home) were investigated as potential predictors of flame retardant exposure.

Among three or more of the flame retardants measured in hand wipes, time since last hand wash, ethnicity, and hours spent at home were associated (*p* < 0.15) with and explained a noticeable proportion of the variance (*R*^2^ > 0.10). These three variables were then included in a multivariable linear model (Suppl. Table 1). Increased time since last hand wash was consistently associated with higher flame retardant levels in bivariate models, but not when adjusting for race/ethnicity and time spent at home. African Americans had lower levels of TCEP (*p* = 0.07), TCIPP (*p* = 0.03), TPHP (*p* = 0.01), and BDE 209 (*p* = 0.04) than Dominican Americans. Women who spend more time outside the home had higher exposure to TCIPP (*p* = 0.10), TDCIPP (*p* = 0.05), TPHP (*p* = 0.09), and ΣAlt-BFRs (*p* = 0.03).

In univariate analysis of urine samples, race/ethnicity was associated with DPHP (*p*=0.03), with African Americans, on average, having higher concentrations of the metabolite. Women who spent more time at home had higher levels of BCIPP (*p*=0.11) in unadjusted models. Generally, there were no predictors of exposure at *p*<0.15 or variables that explained a noticeable proportion of the variance (*R*^2^>0.10) for three or more urinary metabolites (our pre-set criteria). Maternal education had an *R*^2^>0.10 for DPHP (*p*=0.02) and BCIPHIPP (*p*=0.18), with more educated mothers having higher levels of BCIPHIPP but lower levels of DPHP.

### Effectiveness of intervention

#### Hand washing

**Fig. 2:**
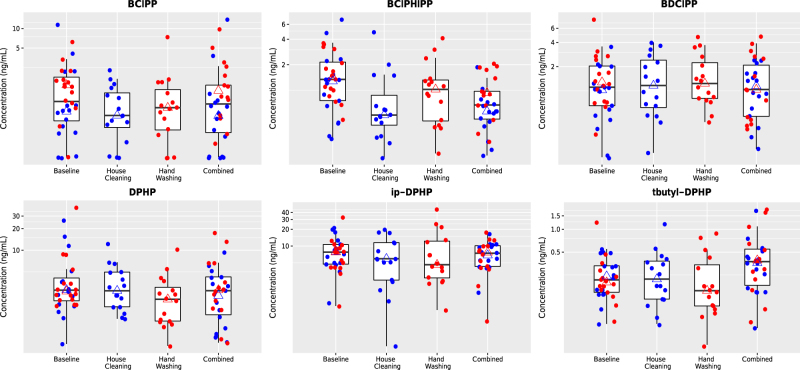
Distributions of urinary metabolites across the study. Boxplots showing the distribution of urinary levels of flame retardant metabolites at baseline, after week 1 (stratified by house cleaning or hand washing intervention), and after week 2 (combined interventions). Boxes represent values between the 25th and 75th percentiles; black lines inside boxes indicate medians; whiskers indicate the range of non-outlier data points. All individual observations are represented by red (hand washing intervention group) or blue (house cleaning intervention group) points. Triangles represent medians for respective group.

**Fig. 3:**
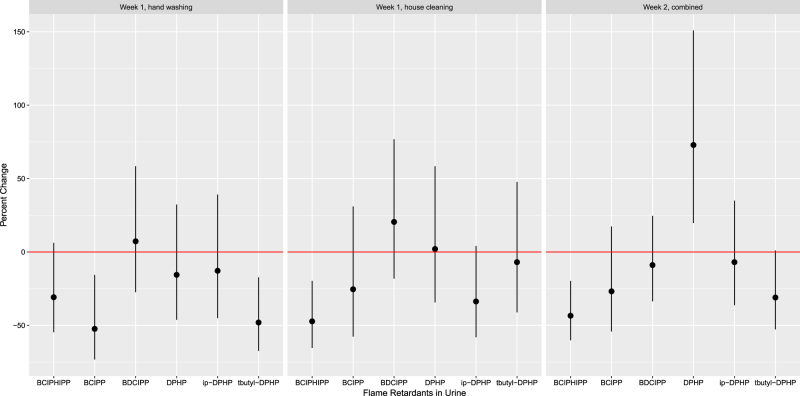
Percent change in urinary metabolites of flame retardants across the study. Percent change and 95% confidence intervals for urinary levels of flame retardant metabolites from baseline to week 1 in each intervention arm (hand washing or house cleaning) and from baseline to week 2 (combined interventions). Points represent percent change from baseline. Error bars represent 95% confidence interval.

We found no statistically significant differences in analytes measured in hand wipes in the hand washing intervention arm after week 1, controlling for time since last hand wash and hours at home (Suppl. Figure 1). We found significant decreases in urinary metabolites (BCIPP, BCIPHIPP, and tbutyl-DPHP) in the hand washing intervention arm after week 1, controlling for hours spent at home (Fig. 2). Figure 3 depicts percent change in urinary metabolites.

After week 1 in the hand washing intervention arm, we found, on average, a 52.36% (95% CI: −73.29, −15.63; *p* = 0.01) decrease in BCIPP; a 30.81% (95% CI: −54.62, 6.18; *p* = 0.09) decrease in urinary metabolites of BCIPHIPP; and a 48.02% (95% CI: −67.37, −17.30; *p* = 0.01) decrease in tbutyl-DPHP (Table 4).

**Table 4:** Fixed effects from multilevel models

	Intervention^*, **^	Percent change	95% Confidence interval	ICC
Hand wipe compound^a^				
TCEP	Week 1, hand washing	−11.340	(−46.74, 46.23)	0.00
	Week 1, house cleaning	−10.61	(−45.66, 47.7)	
	Week 2, combined	−1.51	(−35.6, 50.68)	
TCIPP	Week 1, hand washing	−2.26	(−33.63, 43.33)	0.64
	Week 1, house cleaning	−12.63	(−40.55, 28.4)	
	Week 2, combined	−6.18	(−31.61, 28.4)	
TDCIPP	Week 1, hand washing	35.13	(−9.52, 101.38)	0.61
	Week 1, house cleaning	−3.89	(−35.6, 43.33)	
	Week 2, combined	−8.55	(−34.3, 27.12)	
TPHP	Week 1, hand washing	−6.83	(−46.21, 61.61)	0.61
	Week 1, house cleaning	15.29	(−33.63, 99.37)	
	Week 2, combined	−5.40	(−39.35, 47.7)	
ΣAlt-BFR	Week 1, hand washing	17.76	(−18.94, 71.6)	0.67
	Week 1, house cleaning	−7.04	(−35.6, 34.99)	
	Week 2, combined	5.79	(−21.34, 43.33)	
ΣBDE	Week 1, hand washing	9.21	(−22.12, 53.73)	0.66
	Week 1, house cleaning	1.06	(−28.11, 41.91)	
	Week 2, combined	−7.20	(−29.53, 22.14)	
BDE 209	Week 1, hand washing	−4.97	(−45.66, 64.87)	0.26
	Week 1, house cleaning	−21.65	(−54.62, 34.99)	
	Week 2, combined	−21.16	(−49.84, 24.61)	
Urinary analyte^b^				
BCIPP	Week 1, hand washing**	−52.36	(−73.29, −15.63)	0.45
	Week 1, house cleaning	−25.38	(−57.68, 31)	
	Week 2, combined	−26.79	(−54.16, 17.35)	
DPHP	Week 1, hand washing	−15.55	(−46.21, 32.31)	0.27
	Week 1, house cleaning	2.08	(−34.3, 58.41)	
	Week 2, combined**	72.91	(19.72, 150.93)	
BDCIPP	Week 1, hand washing	7.30	(−27.39, 58.41)	0.52
	Week 1, house cleaning	20.50	(−18.13, 76.83)	
	Week 2, combined	−8.90	(−33.63, 24.61)	
BCIPHIPP	Week 1, hand washing*	−30.81	(−54.62, 6.18)	0.30
	Week 1, house cleaning**	−47.24	(−65.35, −19.75)	
	Week 2, combined**	−43.41	(−60.15, −19.75)	
ip-DPHP	Week 1, hand washing	−12.79	(−45.12, 39.1)	0.41
	Week 1, house cleaning*	−33.69	(−58.1, 4.08)	
	Week 2, combined	−6.90	(−36.24, 34.99)	
tbutyl-DPHP	Week 1, hand washing**	−48.02	(−67.37, −17.3)	0.35
	Week 1, house cleaning	−6.90	(−41.14, 47.7)	
	Week 2, combined*	−31.00	(−52.76, 1.01)	

^a^All hand wipe models control for time since last hand wash and hours at home

^b^All urine models control for hours at home

^*^p-Value <0.10

^**^p-Value <0.05

**Fig. 4:**
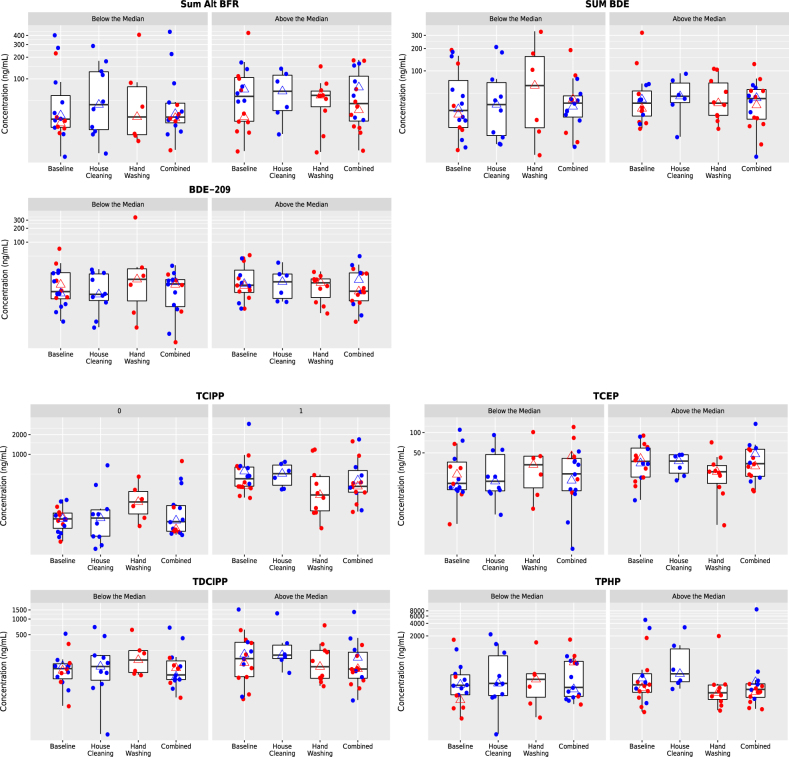
Distributions of flame retardants in hand wipes across the study, stratified by baseline exposure. Boxplots showing the distribution of flame retardant concentrations measured in hand wipes at baseline, after week 1 (stratified by house cleaning or hand washing intervention), and after week 2 (combined interventions), stratified by median level at baseline. Boxes represent values between the 25th and 75th percentiles; black lines inside boxes indicate medians; whiskers indicate the range of non-outlier data points. All individual observations are represented by red (hand washing intervention group) or blue (house cleaning intervention group) points. Triangles represent medians for respective group.

In stratified hand wipe models (Fig. 4), those above median exposure at baseline had consistently lower exposure in the hand washing group across flame retardants after week 1 based on mixed-effects models. Hand washing in highly exposed participants in week 1 was associated with a 56.65% (95% CI: −79.4, −9.52; *p* = 0.03) decrease in TCEP; a 38.63% (95% CI: −64.3, 5.13; *p* = 0.07) decrease in TCIPP; and a 44.61% (95% CI: −69.58, 1.01; *p* = 0.05) decrease in ΣAlt-BFRs (Table 5).

**Table 5:** Fixed effects from multilevel models, stratified by baseline exposure above/below the median

		Above the median		Below the median	
Hand wipe compound^a^					
	Intervention^*, **^	Percent change	95% Confidence interval	Percent change	95% Confidence interval
TCEP	Week 1, hand washing**	−56.65	(−79.4, −9.52)	97.31	(10.52, 252.54)
	Week 1, house cleaning	−44.25	(−74.84, 23.37)	54.98	(−11.31, 169.12)
	Week 2, combined	−36.22	(−66.04, 19.72)	45.44	(−9.52, 133.96)
TCIPP	Week 1, hand washing*	−38.63	(−64.3, 5.13)	81.76	(6.18, 209.57)
	Week 1, house cleaning	−7.66	(−51.81, 76.83)	−7.91	(−41.73, 44.77)
	Week 2, combined	−23.70	(−52.29, 22.14)	19.01	(−22.89, 84.04)
TDCIPP	Week 1, hand washing	−22.71	(−55.07, 32.31)	109.09	(17.35, 274.34)
	Week 1, house cleaning	0.02	(−37.5, 60.00)	−22.78	(−60.54, 52.2)
	Week 2, combined**	−37.10	(−58.93, −2.96)	17.98	(−29.53, 97.39)
TPHP	Week 1, hand washing	−30.29	(−72.75, 78.6)	18.07	(−39.35, 129.33)
	Week 1, house cleaning	1.38	(−51.81, 111.7)	2.64	(−55.96, 138.69)
	Week 2, combined*	−46.98	(−73.82, 7.25)	32.97	(−25.92, 138.69)
ΣAlt-BFR	Week 1, hand washing*	−44.61	(−69.58, 1.01)	84.30	(10.52, 206.49)
	Week 1, house cleaning	−16.35	(−45.12, 27.12)	−17.37	(−58.52, 64.87)
	Week 2, combined	−16.88	(−44.01, 23.37)	16.56	(−26.66, 84.04)
ΣBDE	Week 1, hand washing	11.90	(−37.5, 99.37)	19.58	(−19.75, 78.6)
	Week 1, house cleaning	−16.10	(−50.34, 41.91)	50.58	(−5.82, 141.09)
	Week 2, combined	−15.701	(−46.21, 32.31)	−2.02	(−29.53, 36.34)
BDE 209	Week 1, hand washing	−47.03	(−76.54, 19.72)	79.00	(−12.19, 266.93)
	Week 1, house cleaning**	−57.63	(−81.36, −3.92)	39.10	(−30.23, 177.32)
	Week 2, combined**	−58.63	(−79.4, −16.47)	32.06	(−24.42, 131.64)
		Above the median		Below the median	
Urinary analyte^b^					
	Intervention^*, **^	Percent change	95% Confidence interval	% Change	95% Confidence interval
BCIPP	Week 1, hand washing**	−72.72	(−85.04, −49.34)	42.59	(−48.83, 293.54)
	Week 1, house cleaning**	−77.36	(−90.93, −40.55)	41.84	(−25.17, 169.12)
	Week 2, combined	−34.22	(−59.34, 16.18)	−9.93	(−52.76, 71.6)
DPHP	Week 1, hand washing	−1.79	(−45.12, 84.04)	−24.72	(−61.33, 47.7)
	Week 1, house cleaning	37.08	(−25.92, 156)	−23.51	(−60.15, 46.23)
	Week 2, combined**	140.56	(37.71, 322.07)	31.35	(−22.12, 122.55)
BDCIPP	Week 1, hand washing	−26.85	(−50.34, 17.35)	65.82	(−6.76, 194.47)
	Week 1, house cleaning	−28.44	(−59.34, 25.86)	95.345	(23.37, 209.57)
	Week 2, combined**	−36.76	(−55.07, −3.92)	29.78	(−13.93, 97.39)
BCIPHIPP	Week 1, hand washing**	−46.85	(−63.21, −17.3)	−11.42	(−55.96, 78.6)
	Week 1, house cleaning**	−74.31	(−83.47, −58.1)	−5.88	(−50.34, 78.6)
	Week 2, combined**	−62.38	(−72.75, −45.12)	−21.85	(−55.51, 36.34)
ip-DPHP	Week 1, hand washing	−33.10	(−59.34, 17.35)	10.94	(−47.8, 133.96)
	Week 1, house cleaning**	−52.01	(−69.88, −16.47)	−6.19	(−55.07, 95.42)
	Week 2, combined	−27.68	(−50.34, 15.03)	17.36	(−36.24, 115.98)
tbutyl-DPHP	Week 1, hand washing**	−56.28	(−77.69, −10.42)	−40.60	(−69.88, 16.18)
	Week 1, house cleaning	−16.65	(−55.07, 58.41)	9.06	(−47.27, 124.79)
	Week 2, combined*	32.02	(−66.71, 4.08)	−20.16	(−54.62, 40.49)

^a^All hand wipe models control for time since last hand wash and hours at home

^b^All urine models control for hours at home

*p-Value <0.10

**p-Value <0.05

#### House cleaning

We found no statistically significant differences in analytes measured in hand wipes in the house cleaning intervention arm after week 1, controlling for time since last hand wash and hours at home (Suppl. Figure 1). We found significant decreases in urinary metabolites (BCIPHIPP and ip-DPHP) in the house cleaning intervention arm after week 1, controlling for hours spent at home (Fig. 2).

After week 1 in the house cleaning intervention arm, we observed, on average, a 47.24% (95% CI: −65.35, −19.75; *p* = 0.003) decrease in BCIPHIPP and a 33.69% (95% CI: −58.1, 4.08; *p* = 0.08) decrease in ip-DPHP (Table 4).

In stratified hand wipe models (Fig. 4), those above median exposure at baseline had consistently lower exposure in the house cleaning intervention arm across flame retardants after week 1 based on mixed-effects models. In those most exposed, house cleaning in week 1 was associated with a 57.63% (95% CI: −81.36, −3.92; *p* = 0.04) decrease in BDE 209 (Table 5). In stratified models of urinary metabolites, in addition to flame retardants with significant reductions prior to stratification (BCIPHIPP and ip-DPHP), BCIPP decreased 77.36% (95% CI: −90.93, −40.55, *p* = 0.004) after week 1 of house cleaning (Suppl. Figure 2).

#### Combined

We found no statistically significant differences in analytes measured in hand wipes after week 2 of combined interventions, controlling for time since last hand wash and hours at home (Suppl. Figure 1). We found significant decreases in urinary metabolites (BCIPHIPP and tbutyl-DPHP) after week 2 of combined interventions, controlling for hours spent at home (Fig. 2).

After week 2 of combined hand washing and house cleaning, we found a 43.41% (95% CI: −60.15, −19.75; *p* = 0.002) decrease in BCIPHIPP; a 31.00% (95% CI: −52.76, 1.01; *p* = 0.06) decrease in tbutyl-DPHP; and an unexpected 72.91% (95% CI: 19.72, 150.93; *p* = 0.004) increase in DPHP (Table 4).

In stratified hand wipe models (Fig. 4), those above median exposure at baseline had consistently lower exposure after week 2 across flame retardants. After week 2 of combined interventions, those above the median at baseline had TDCIPP levels 37.10% (95% CI: −58.93, −2.96; *p* = 0.04) lower than at baseline; TPHP levels 46.98% (95% CI: −73.82, 7.25; *p* = 0.08) lower than at baseline; and BDE 209 levels 58.63% (95% CI: −79.4, −16.47; *p* = 0.02) lower than at baseline (Table 5). In stratified models of urinary metabolites, in addition to flame retardants with significant reductions prior to stratification (BCIPHIPP and tbutyl-DPHP), BDCIPP decreased 36.76% (95% CI: −55.07, −3.92; *p* = 0.03) after combined interventions in week 2 (Suppl. Figure 2).

#### Intra-class correlations

We used variance estimates from the mixed-effects models to measure ICCs among observations within the same individual (Table 4), estimating the percentage of the total variance explained. Intra-individual correlations in urine samples ranged from 0.27 (DPHP) to 0.52 (BDCIPP). In hand wipes, the lowest ICC observed was 0.00 (TCEP), but five of the seven analytes had ICCs between 0.61 (TDCIPP and TPHP) and 0.67 (ΣAlt-BFR).

#### Cross-sample correlations

We investigated Spearman correlations between parent compounds in hand wipes and urinary metabolites. Consistent correlations in mothers were found between averaged hand wipes and averaged urine samples (baseline, week 1, and week 2); sample pooling across the length of the study was done to stabilize intra-individual variation in exposure and metabolism (Table 6).

**Table 6:** Correlation in mothers between flame retardants in hand wipes and urinary metabolites

Averaged over week 1					Averaged over week 2				
	BCIPP	DPHP	BDCIPP	BCIPHIPP		BCIPP	DPHP	BDCIPP	BCIPHIPP
TCIPP	0.38	−0.08	0.08	−0.13	TCIPP	0.088	0.138	0.226	−0.242
	(0.03)	(0.65)	(0.67)	(0.47)		(0.65)	(0.48)	(0.24)	(0.21)
TDCIPP	−0.36	−0.03	0.41	−0.05	TDCIPP	−0.295	0.114	0.549	−0.110
	(0.05)	(0.87)	(0.02)	(0.79)		(0.12)	(0.56)	(0.002)	(0.57)
TPHP	−0.23	0.42	−0.10	0.22	TPHP	−0.181	0.443	−0.003	0.158
	(0.21)	(0.02)	(0.59)	(0.24)		(0.35)	(0.02)	(0.99)	(0.41)
*Throughout the study*									
	BCIPP	DPHP	BDCIPP	BCIPHIPP					
TCIPP	0.26	0.07	0.13	−0.04					
	(0.18)	(0.72)	(0.50)	(0.84)					
TDCIPP	−0.41	0.15	0.51	−0.11					
	(0.03)	(0.46)	(0.01)	(0.57)					
TPHP	−0.27	0.51	0.00	0.29					
	(0.16)	(0.01)	(0.99)	(0.13)					

*p*-Value in parentheses

TDCIPP in maternal hand wipes was significantly correlated with BDCIPP in urine (*r* = 0.51; *p* = 0.01). TPHP in hand wipes was significantly correlated with DPHP (*r* = 0.51; *p* = 0.01). Correlations between TDCIPP and TPHP and their metabolites within week 1 and week 2 appeared similar. TCIPP concentrations were not significantly correlated with urinary BCIPP (*r* = 0.26; *p* = 0.18) over the course of the study, but they were during week 1 (*r* = 0.38; *p* = 0.03); TCIPP was consistently non-significantly and negatively correlated with BCIPHIPP.

#### Sensitivity analyses

Analysis using unadjusted Wilcoxon signed rank tests for observations across time produced similar, though attenuated, results. We found no significant differences in concentrations between time points in hand wipe samples. In urine samples stratified by intervention group, there were significant decreases in BCIPP (*p* = 0.02) and BCIPHIPP (*p* = 0.04), both metabolites of TCIPP, within the hand washing group between baseline and week 1. We found no significant differences within the house cleaning group between baseline and week 1. Between baseline and week 2, we found a significant decrease in BCIPHIPP (*p* = 0.0003) and a significant increase in tbutyl-DPHP (*p* = 0.02). In adjusted models, the difference between baseline and week 2 tbutyl-DPHP was negative and significant. The significant increase in DPHP after week 2 seen in the mixed-effects model was not replicated using a Wilcoxon sign ranked test.

#### Simulations

We conducted simulations using hand wipe and urine data to determine if our stratified results were subject to modeling choices and to assess the models’ robustness (Suppl. Figures 3 and 4). Simulations of hand wipe data above the median at baseline confirmed that negative beta coefficients for TCEP at all time points, TDCIPP after week 2, and BDE 209 at all time points were more extreme than chance. Coefficients for TCIPP, TPHP, and ΣAlt-BFR, while at least marginally significant in stratified models, could not be distinguished from random chance. Simulations of urine data above the median at baseline upheld that decreases in BCIPP after week 1 of house cleaning and in BDCIPP after week 2 (the only significant associations in the stratified models that were not significant in the original models) were more extreme than chance findings. All significant coefficients in the stratified urine models (including those significant in the original models) were verified by simulations.

#### Compliance

Participants answered questionnaires concerning compliance following each intervention week. Twenty participants (62.5%) responded that their house cleaning habits had changed over the course of the intervention. Five women (15.6%) said that they spent less time, nine (28.1%) said that they spent about the same amount of time, and 17 (53.1%) said they spent more time cleaning during the intervention than usual. Only five women (15.6%) had a vacuum available for their use at baseline. Twenty-eight (87.5%) used the vacuum they were given as incentive at least once.

When asked directly if their hand washing habits had changed, 22 participants (68.8%) responded that they had. When comparing reported frequency from baseline to the end of the week of their hand washing intervention, 16 women (50%) reported increases, nine (28.1%) reported decreases, and seven (21.9%) reported no change in the number of times they washed their hands per day during the previous week. This does not consider duration or manner of hand washing. Restricting analyses to participants who complied with intervention protocol did not change the direction or magnitude of results.

## Discussion

This is the first study to assess changes in flame retardant exposure as the result of house cleaning and hand washing practices. The results of our case-crossover design support the hypothesis that hand washing and house cleaning can reduce exposure to some, though not all, flame retardants measured through dermal exposure and urinary metabolites. In hand wipes, reductions in exposure were only found in those individuals with exposure above the median at baseline, indicating that behavioral intervention may not be effective for those with initially low exposure. In urine samples, consistent, substantial reductions in exposure across the majority of metabolites were found in the original, unstratified sample, but an unexpected increase in DPHP, a metabolite of TPHP, was found after week 2 of combined exposure.

Baseline OPFR concentrations in hand wipes and metabolites in urine were higher in our participants than in an exposure assessment conducted on adults over the age of 18 in North Carolina [20]. Compared to a study on mother and child pairs also in North Carolina, our mothers had higher urinary levels of BCIPP, DPHP, ip-DPHP, and tbutyl-DPHP, but lower levels of BDCIPP [25]. Our study also found higher levels of BCIPP and DPHP, but lower levels of BCIPHIPP and BDCIPP in mothers than a study of mothers and children (between 2 and 70 months old) in California [48]. Participants in the latter two studies were predominantly White, and previous studies have found higher body burdens of PBDEs among non-White women compared to White women [44]. Thus, our observation of racial/ethnic differences in exposure levels supports previous findings.

Recent studies have reported seasonal variation in urinary OPFR metabolite concentrations, with highest levels observed in the summer [49]. Our intervention took place between December and May, and Hoffman et al. found significant differences between levels of BDCIPP in the winter and spring. While temporal differences may affect the concentrations of urinary metabolites measured in our study, they will not influence the percent change within an individual, thus should not bias our results concerning the effectiveness of the intervention.

Baseline exposure in our study was predicted by race/ethnicity, time since last hand wash (in hand wipe samples only), and hours spent at home. African American mothers in our study had lower levels of OPFRs and BDE 209 than Dominicans, though other studies have shown Dominicans to have lower levels of summed PBDEs (children only), BDE 209, and Alt-BFRs, presumably due to cultural differences in cleaning practices [24]. Time outside the home, in unspecified locations where women were working (thus, not in control of the environment), predicted increased OPFR and Alt-BFR exposure. The positive association between time outside the home and flame retardant exposure challenges the effectiveness of a house cleaning-based intervention.

After 1 week of hand washing, we found significant decreases in levels of BCIPP, BCIPHIPP, and tbutyl-DPHP in urine samples. And while no decreases were found in hand wipes from the entire study sample, hand washing after week 1 in those highly exposed at baseline was significantly associated with decreases in TCEP, TCIPP, and ΣAlt-BFRs in hand wipes. Simulations corroborated results for TCEP, but not for TCIPP or ΣAlt-BFRs, meaning that TCEP levels were unlikely to have declined as a statistical byproduct of regression. BCIPP and BCIPHIPP are both metabolites of TCIPP [48], thus the significant findings in parent compounds and in urinary metabolites support the plausibility of the effectiveness of hand washing as preventative of TCIPP exposure. We did not measure metabolites of TCEP or ΣAlt-BFRs in this study.

After 1 week of house cleaning, we found significant decreases in levels of BCIPHIPP and ip-DPHP in urine samples. Restricted to those above the median at baseline, house cleaning after week 1 was significantly associated with decreased BDE 209 in hand wipes, which was supported by simulations. TCIPP, the parent of BCIPHIPP, was reduced, but not significantly, by house cleaning in this study. Neither the parent of ip-DPHP nor a metabolite of BDE 209 were measured.

After week 2 of combined interventions, we found significant decreases in levels of BCIPHIPP and tbutyl-DPHP and an unexpected significant increase in DPHP in urine samples. Combined house cleaning and hand washing during week 2 in those more exposed at baseline was associated with lower levels of TDCIPP, TPHP, and BDE 209 in hand wipes. Only the effect on BDE 209 was validated by simulations. TCIPP, the parent of BCIPHIPP, and TDCIPP’s metabolite, BDCIPP, were reduced, but not significantly, by combined interventions in this study.

Results for TPHP and its metabolite DPHP appear contradictory. Even stratified to those most exposed at baseline, combined house cleaning and hand washing during week 2 led to a significant decrease in dermal TPHP exposure but an increase in urinary levels of DPHP. It is known that chemicals other than TPHP (including monosubstituted isopropylated triaryl phosphate (mono-ITP), 2-ethylhexyl diphenyl phosphate (EHDPHP), and isodecyl diphenyl phosphate (id-DPHP)) may metabolize to form DPHP [20]. Thus, it is possible that, though the intervention was effective in reducing TPHP exposure, it unintentionally led to increased exposure to one or more of the other possible parent compounds of DPHP. Results for TCIPP and BCIPP, while in the same direction, may differ in magnitude as urinary levels of BCIPP are limited by the low formation yield of BCIPP from TCIPP, as TCIPP has been shown to be metabolized to a dechlorinated carboxylic acid metabolite more often than to the dialkyl ester [25]. Effect of intervention on all parent–metabolite pairs (TDCIPP and BDCIPP, TPHP and DPHP, TCIPP and BCIPP, and TCIPP and BCIPHIPP) may disagree due to the limitation of the spot urine sample. Since sample collection was not conducted in a standardized way, rather at the convenience of study participants, disagreement may also reflect unmeasured behaviors and not differences in the effectiveness of the intervention.

It is possible to conceive of time spent at home as an effect modifier instead of a confounder, as a cleaning intervention to reduce flame retardant exposure may be more effective for individuals who spend more time at home. Unfortunately, we did not have the statistical power to evaluate this scenario. Examination of models stratified by more/less time spent at home did not suggest a difference in the effect of the intervention.

Limitations of this study include its small sample size, generalizability of the cohort, multiple comparisons, and the imperfections of spot urine samples. Because of our small sample size, we cannot rule out chance findings despite statistical significance of our effect estimates. The intervention’s case-crossover design, however, where individuals act as their own controls, removes the possible time-invariant confounding factors of cross-sectional studies. Additionally, the small sample size allowed for more robust data collection, including hand wipes, urine samples, detailed questionnaires, wristbands (included in a companion article), and house dust (not included in the present analysis). Generalizability from this sample to the U.S. population is limited because of the purposeful sampling of African American and Dominican families. As minority residents of urban environments face a disproportionate burden from environmental toxicants, participants from the Sibling-Hermanos cohort were intentionally selected to address concerns relevant to minority health and living conditions. We analyzed seven flame retardants in hand wipes and six metabolites in urine, begging the question of multiple comparisons. In this regard, we looked for consistency in magnitude and direction of effect estimates. Finally, spot urine samples, which do not account for intra-individual variations in analyte levels, were used to analyze OPFR metabolites. We unfortunately do not have information about the length of time spent at home prior to sample collection, which would better characterize exposure. Since OPFRs have short half-lives, spot urine samples may have introduced outcome misclassification. However, this misclassification of metabolite level would be random and not associated with exposure (i.e., intervention arm), and thus, would be non-differential, biasing our results toward the null.

Though neither intervention arm nor combined hand washing and house cleaning led to a reduction across all flame retardants, four of the six urinary metabolites measured (all but BDCIPP and DPHP) decreased (at least marginally) significantly after week 1 or week 2. In participants above the median at baseline, six of the seven flame retardants measured (all but ΣBDE) decreased after week 1 or week 2. The simulations show that reductions in TCEP and BDE 209 were unlikely to be byproducts of regression to the mean. These results imply that both hand washing and house cleaning can be effective methods of exposure reduction to flame retardants. This evidence supports the EPA’s recommendations of house cleaning and hand washing, with the qualification that a substantial proportion of our participants’ exposure came from outside the home, where cleaning may not be an option for exposure mitigation.

This intervention took place over the course of 2 weeks, requiring a sustained behavioral change over the course of the study. Twenty-two of the 32 mothers reported that their (and their children’s) hand washing behavior did, in fact, change as a result of the intervention, while 20 mothers reported vacuuming more often. After 2 weeks with the addition of the second intervention, there is a possibility that participants could not maintain the study’s recommendations. In addition, exposure likely occurs in places other than the home, such as places of work and transportation modalities where increased cleaning might also be effective in reducing exposure. However, while hand washing is not specific to the home, individuals may or may not be able to control the cleanliness of the work places or transit methods. This study does not address how much individuals need to clean their homes or wash their hands to make a difference with regard to flame retardant exposure. As a sustained behavioral change is difficult, it is necessary to give practical and achievable recommendations.

## Conclusion

One week of increased hand washing or targeted house cleaning is enough, in some cases, to reduce exposure to flame retardants by half. Results of this study suggest that behavioral interventions can significantly decrease exposure levels of some, but not all, flame retardants. None of the reported flame retardants were reduced below the MDL, indicating that individual behavior cannot entirely mitigate exposure. As participants faced additional exposure outside the home, house cleaning and hand washing can help to reduce, but not eliminate, exposure to flame retardants.

## Electronic supplementary material

Supplemental Materials
