# Supplementary material for: Flame retardant exposure assessment: findings from a behavioral intervention study
Source: J Expo Sci Environ Epidemiol. 2018 Jun 28;29(1):33–48. doi: 10.1038/s41370-018-0049-6 (PMC6460909; doi:10.1038/s41370-018-0049-6)
Supplement: Supplementary file 1 — Supplemental Materials [file 41370_2018_49_MOESM1_ESM.docx]

**Supplemental Materials.**

**Supplementary Figure 1. Distributions of flame retardant concentrations in hand wipes across the study.**


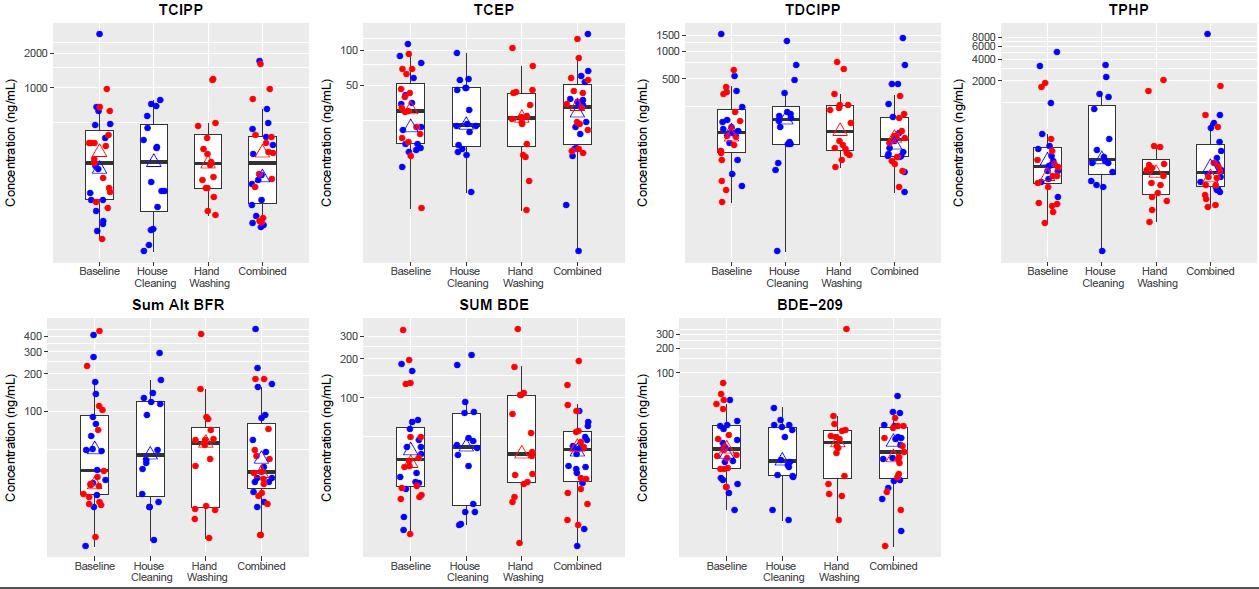
Boxplots showing the distribution of flame retardant concentrations measured in hand wipes at baseline, after week 1 (stratified by house cleaning or hand washing intervention), and after week 2 (combined interventions). Boxes represent values between the 25th and 75th percentiles; black lines inside boxes indicate medians; whiskers indicate the range of nonoutlier data points. All individual observations are represented by red (hand washing intervention group) or blue (house cleaning intervention group). Triangles represent medians for respective group.

**Supplementary Figure 2. Distributions of urinary metabolites across the study, stratified by baseline exposure.**


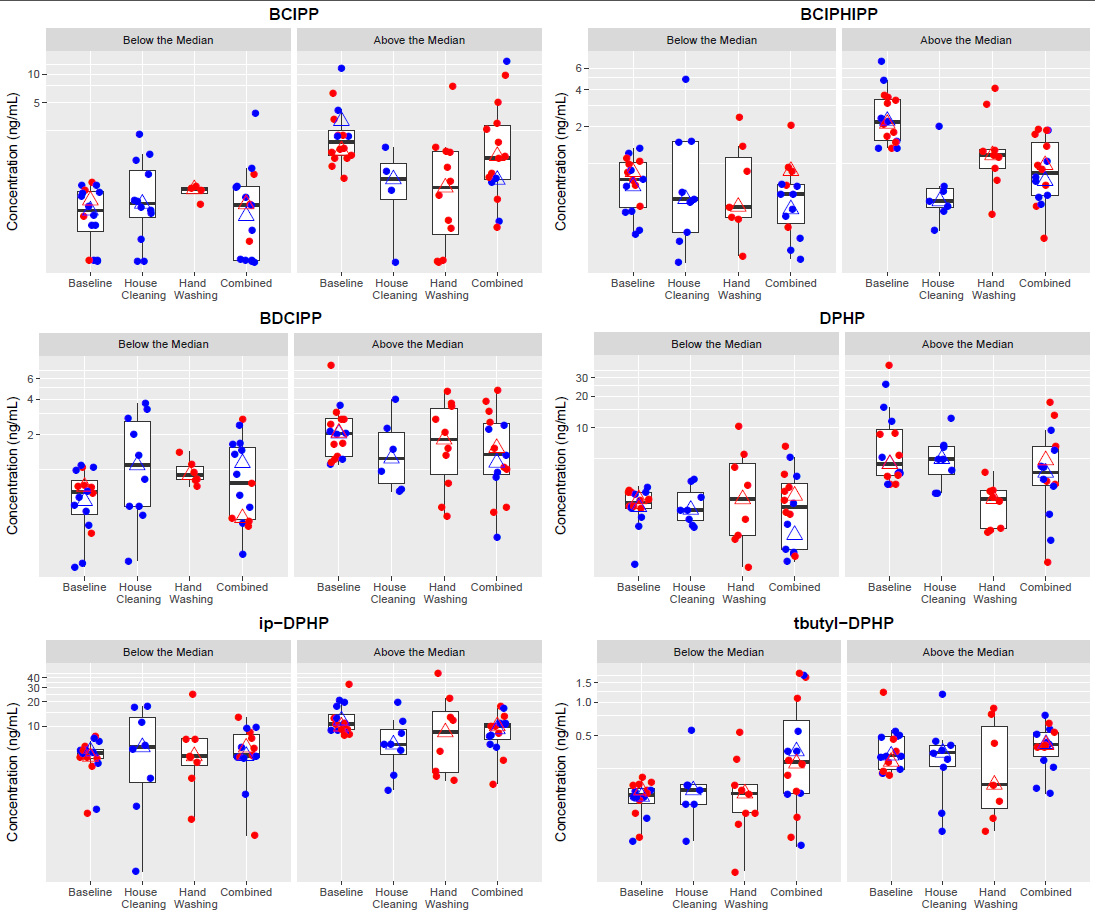


Boxplots showing the distribution of urinary levels of flame retardant metabolites at baseline, after week 1 (stratified by house cleaning or hand washing intervention), and after week 2 (combined interventions), stratified by median level at baseline. Boxes represent values between the 25th and 75th percentiles; black lines inside boxes indicate medians; whiskers indicate the range of nonoutlier data points. All individual observations are represented by red (hand washing intervention group) or blue (house cleaning intervention group). Triangles represent medians for respective group.

**Supplementary Figure 3. Simulations of mixed-effect models of hand wipe measures, stratified by baseline exposure.**


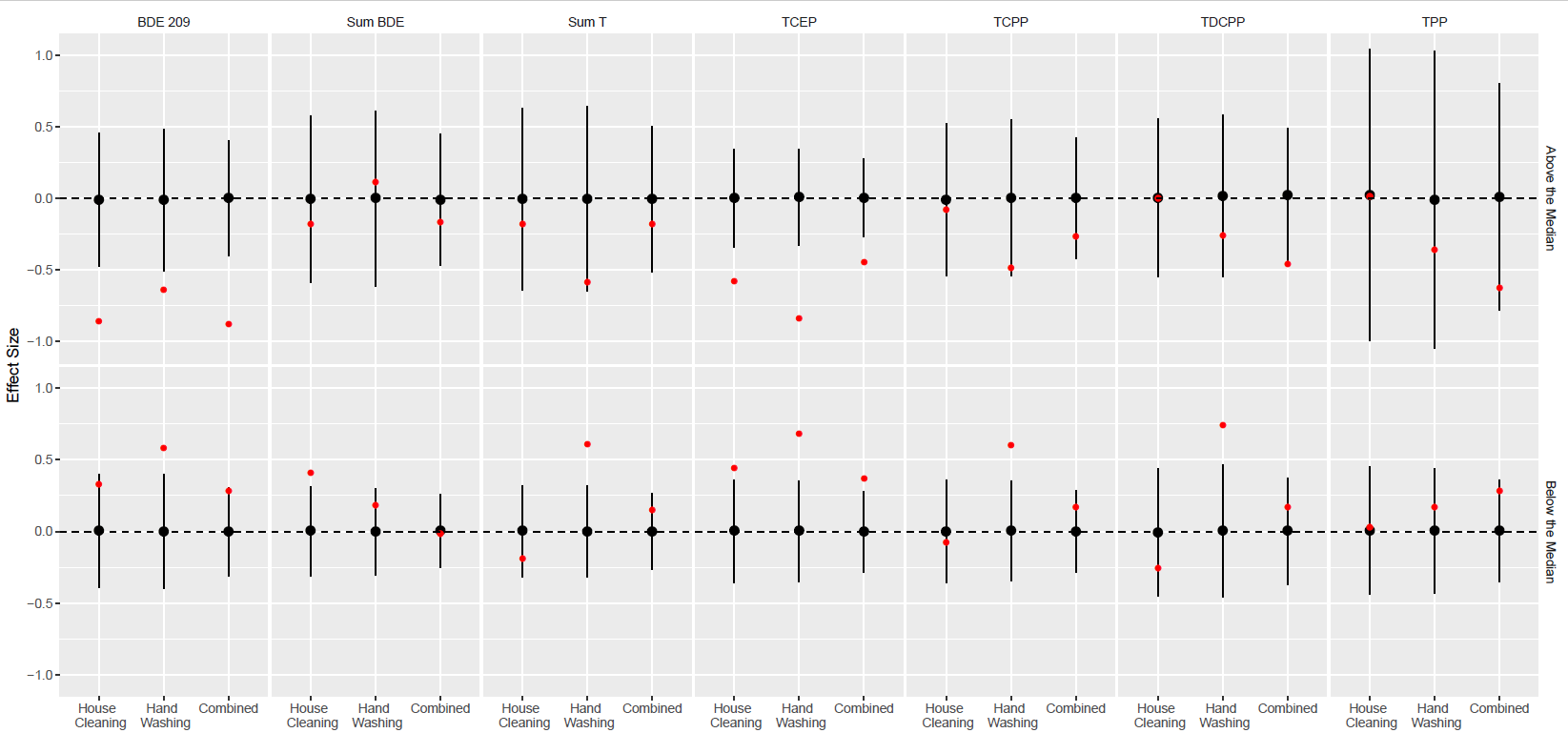


Plot shows the distribution of simulated beta coefficients (mean and 95% confident interval in black) compared to observed beta coefficients in red (3 points represent effect estimates for house cleaning vs. baseline, hand washing vs. baseline, and combined intervention vs. baseline) for each of seven measured flame retardants in hand wipes, stratified by median value at baseline.

**Supplementary Figure 4. Simulations of mixed-effect models of urinary metabolites, stratified by baseline exposure.**


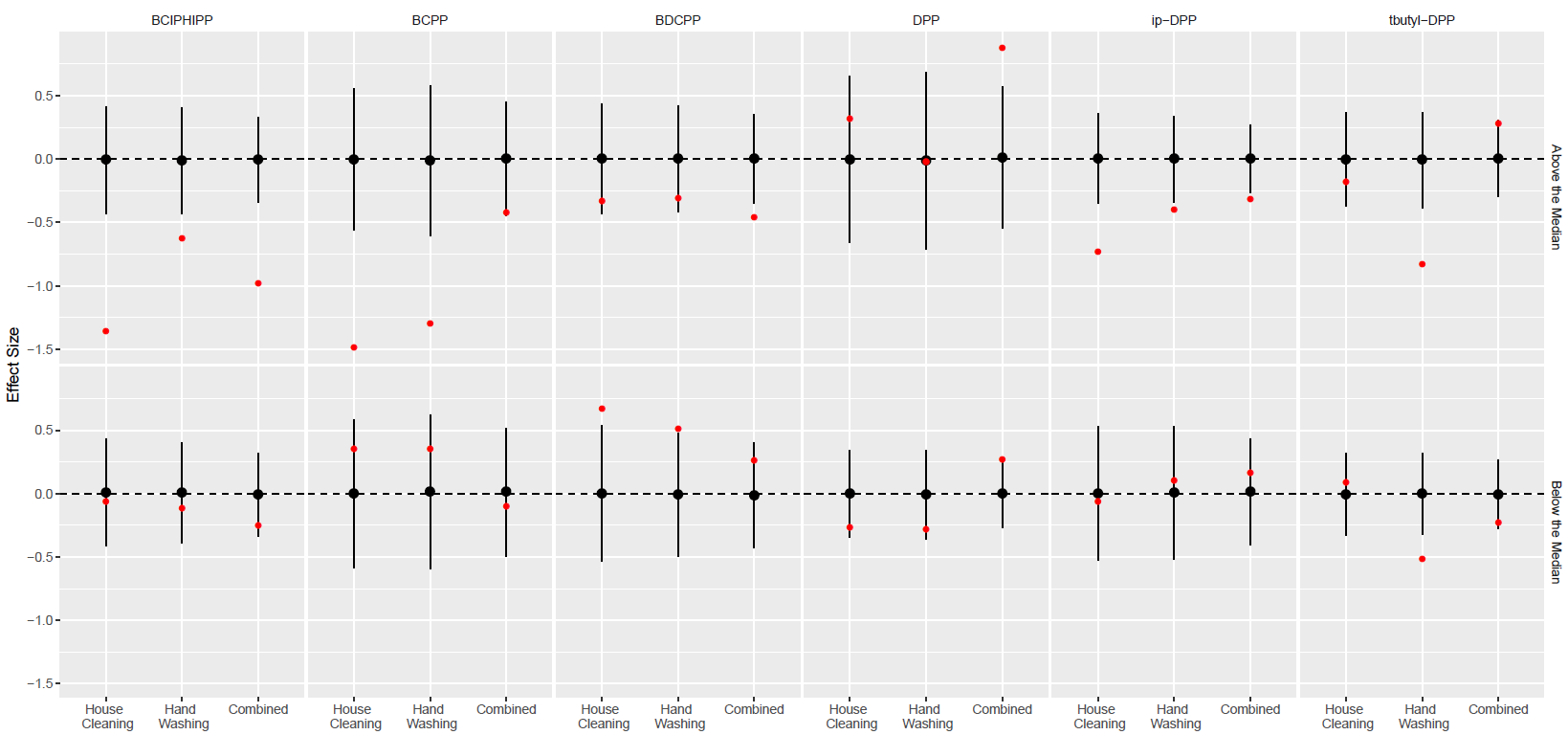


Plot shows the distribution of simulated beta coefficients (mean and 95% confident interval in black) compared to observed beta coefficients in red (3 points represent effect estimates for house cleaning vs. baseline, hand washing vs. baseline, and combined intervention vs. baseline) for each of six OPFR metabolites, stratified by median value at baseline.

**Supplementary Table 1. Risk factors for flame retardant exposure in handwipes at baseline.**

|  | Last Hand Wash^1^ | | Ethnicity^2^ | | Hours at Home | | Total Model  R^2^ |
| --- | --- | --- | --- | --- | --- | --- | --- |
|  | Beta | P-value | Beta | P-value | Beta | P-value |  |
| TCEP | -0.09 | 0.41 | -0.52 | 0.07 | -0.25 | 0.37 | 0.16 |
| TCIPP | 0.08 | 0.52 | -0.75 | 0.03 | 0.58 | 0.10 | 0.24 |
| TDCIPP | 0.13 | 0.32 | -0.46 | 0.18 | -0.70 | 0.05 | 0.22 |
| TIPP | 0.25 | 0.12 | -1.25 | 0.01 | -0.75 | 0.09 | 0.37 |
| ΣAlt-BFR | 0.21 | 0.13 | -0.40 | 0.26 | -0.78 | 0.03 | 0.25 |
| ΣBDE | 0.23 | 0.07 | 0.09 | 0.79 | -0.38 | 0.25 | 0.15 |
| BDE 209 | 0.22 | 0.07 | -0.65 | 0.04 | -0.08 | 0.80 | 0.23 |

^1^ Beta represents change per hour since last hand wash.

^2^ Beta represents difference between African American and Dominican (reference) mothers.

**Instructional Materials.**

Participating mothers were provided verbal instructions and postcard reminders with the following information.

**Postcard FRONT [both cards]:**

Chemicals called flame-retardants were added to foam in furniture and other products, including electronics to meet a fire safety law.

Flame-retardants can escape from products and end up in household dust.

In this study, we are trying to see if washing hands frequently and cleaning the house can reduce families’ contact with these chemicals.

**Postcard #1 (Handwashing) BACK:**

**What we will do today:**

• Use some wipes to collect dust that you cannot see from your hands and the hands of your child

• Collect a urine sample from you and your child

**What we are asking you to do this week:**

• Try to wash your hands more often, particularly before you eat

• Try to encourage your child to wash his/her hands more often, particularly before he/she eats

**Postcard #2 (Cleaning) BACK:**

**What we will do today:**

• Use some wipes to collect dust that you cannot see from your hands and the hands of your child

• Collect a urine sample from you and your child

**What we are asking you to do this week:**

• Try to remove dust from around your house

• Use the vacuum as much as you like; in general, we suggest that you open your windows when you use the vacuum to reduce your exposure to recirculating dust. Please don’t empty the dust canister in your vacuum; we will do it for you next week and will replace it with a clean one.
